# One Bacterial Cell, One Complete Genome

**DOI:** 10.1371/journal.pone.0010314

**Published:** 2010-04-23

**Authors:** Tanja Woyke, Damon Tighe, Konstantinos Mavromatis, Alicia Clum, Alex Copeland, Wendy Schackwitz, Alla Lapidus, Dongying Wu, John P. McCutcheon, Bradon R. McDonald, Nancy A. Moran, James Bristow, Jan-Fang Cheng

**Affiliations:** 1 Department of Energy Joint Genome Institute, Walnut Creek, California, United States of America; 2 Department of Ecology and Evolutionary Biology, University of Arizona, Tucson, Arizona, United States of America; University of Hyderabad, India

## Abstract

While the bulk of the finished microbial genomes sequenced to date are derived from cultured bacterial and archaeal representatives, the vast majority of microorganisms elude current culturing attempts, severely limiting the ability to recover complete or even partial genomes from these environmental species. Single cell genomics is a novel culture-independent approach, which enables access to the genetic material of an individual cell. No single cell genome has to our knowledge been closed and finished to date. Here we report the completed genome from an uncultured single cell of *Candidatus* Sulcia muelleri DMIN. Digital PCR on single symbiont cells isolated from the bacteriome of the green sharpshooter *Draeculacephala minerva* bacteriome allowed us to assess that this bacteria is polyploid with genome copies ranging from approximately 200–900 per cell, making it a most suitable target for single cell finishing efforts. For single cell shotgun sequencing, an individual *Sulcia* cell was isolated and whole genome amplified by multiple displacement amplification (MDA). Sanger-based finishing methods allowed us to close the genome. To verify the correctness of our single cell genome and exclude MDA-derived artifacts, we independently shotgun sequenced and assembled the *Sulcia* genome from pooled bacteriomes using a metagenomic approach, yielding a nearly identical genome. Four variations we detected appear to be genuine biological differences between the two samples. Comparison of the single cell genome with bacteriome metagenomic sequence data detected two single nucleotide polymorphisms (SNPs), indicating extremely low genetic diversity within a *Sulcia* population. This study demonstrates the power of single cell genomics to generate a complete, high quality, non-composite reference genome within an environmental sample, which can be used for population genetic analyzes.

## Introduction

Microorganisms on Earth have undergone an estimated 3.8 billion years of evolution and comprise the vast majority of biological diversity. The characterization of these life forms not only aids our understanding of genetic and physiological diversity, community ecology and biogeochemistry, but also furthers the development of novel compounds and processes for biotechnology, pharmaceuticals and other applications and industries. As only a minute fraction of microbial species are estimated to grow using current culturing techniques [1], culture-independent methods are crucial. Metagenomics [1] and, more recently, single cell genomics [2], [3], [4] have become the methods of choice to access the genetic material of the uncultured microbial majority to enable predictions and drive hypothesis about the life-style of these species as based on their coding potential. Metagenomics has provided the first glimpse into the life of uncultured microorganisms [5], a breakthrough that not only led to a large array of new gene discoveries [6], [7], [8], but also enabled close-to-complete [9], [10] and complete [11], [12] genome access of various uncultured microbes. In highly diverse environments, however, notable genome assembly for given community members is likely not feasible, or only at the very high cost of deep sequencing. Moreover, heterogeneity within complex environmental samples can pose a major challenge during sequence assembly. The more recently developed single cell genomic approach allows the genome analysis of individual community members, largely independent of the complexity of the sample environment [4], [13]. Single cells can be isolated from the environment using optical tweezers, micromanipulation, FACS, serial dilutions, microfluidic chips or laser capture microdissection. After cell lysis, the microbial genome is amplified using multiple displacement amplification (MDA) [4], [14], enabling random genome shotgun sequencing. Several uncultured single microbial genomes have recently been sequenced using the single cell approach [15], [16], [17], [18]. While the recovery of >0.5 Mb large contigs has been demonstrated for some of these single amplified genomes (SAGs), no single cell genome has to our knowledge been closed and finished to date.

In this study, we applied single cell genomics to recover the complete *Candidatus* Sulcia muelleri DMIN (hereafter simply referred to as *Sulcia*) genome from the green sharpshooter (GSS) *Draeculacephala minerva* Ball (Insecta: Hemiptera: Cicadellidae). GSS are a prominent group of sap-feeding leafhoppers that are involved in the spread of the plant pathogen *Xylella fastidiosa*, which causes a number of serious plant diseases [19], [20]. Sharpshooters are inhabited by two obligate bacterial symbionts, the Gammaproteobacterial *Candidatus* Baumannia cicadellinicola [21] and *Sulcia*, a Bacteroidetes [22]. Vertically transmitted via eggs, they are housed in a specialized host organ called a bacteriome. Besides inhabiting leafhoppers, *Sulcia* has been found in a wide range of additional related insect hosts, such as treehoppers, cicadas, spittlebugs, and planthoppers, representing an ancient symbiont, that was most likely acquired by an ancient shared ancestor of these related insect hosts [21], [22]. The genome of *Sulcia* from the glassy-winged sharpshooter (GWSS) *Homalodisca vitripennis* and the cicada *Diceroprocta semicincta* have previously been sequenced using metagenomics [23], [24], [25]. In both cases, the metagenomic data were dominated by contaminating host reads, and therefore to obtain complete *Sulcia* genomes the samples needed to be ‘oversequenced’ to a large degree [24], [25].

Here, we report the recovery of the complete circular *Sulcia* genome from a single polyploid bacterial cell derived from the GSS bacteriome. To validate the correctness of the single cell genome, we independently reconstructed the *Sulcia* genome using metagenomics, yielding a nearly identical genome. The variations we detected are likely of true biological origin. To evaluate the genetic diversity within the *Sulcia* community, metagenomic sequence data were generated and aligned to the finished single cell derived reference. We were able to locate two single nucleotide polymorphisms (SNPs) within the metagenomic data, indicating that there is very low genetic diversity within the bacteriome *Sulcia* community within the sample insect population.

## Results and Discussion

### Polyploidy in *Sulcia*

To assess whether complete sequencing from a single uncultured bacterial cell is feasible, we aimed to sequence a polyploid genome. The presence of multiple genome copies could enable finishing a single cell genome, even if double-stranded DNA breaks were introduced during the single cell lysis step and handling for whole genome amplification. While polyploidy has been reported in the intracellular aphid symbiont *Buchnera* [26], [27], it was only suspected for *Sulcia*, based on the intensity of DNA staining in the very large *Sulcia* cells. In pea aphid hosts, *Buchnera* has been shown to average 20 to several hundred genome copies per cell, depending on the developmental stage and morph of the host. We used digital PCR [28] to determine if *Sulcia* has a polyploid genome. The genome copy number within four individual *Sulcia* cells were assayed using two genomic loci, resulting in estimations of 180–880 genome copies per cell (Figures S1). We counted 180 genome copies for cell 1 (both loci), 140 (locus A) and 240 (locus B) genome copies for cell 2, 740 (locus A) and 880 (locus B) genome copies for cell 3; and 480 genome copies for cell 4 (both loci).

### Single cell genome reconstruction

**Figure 1:**
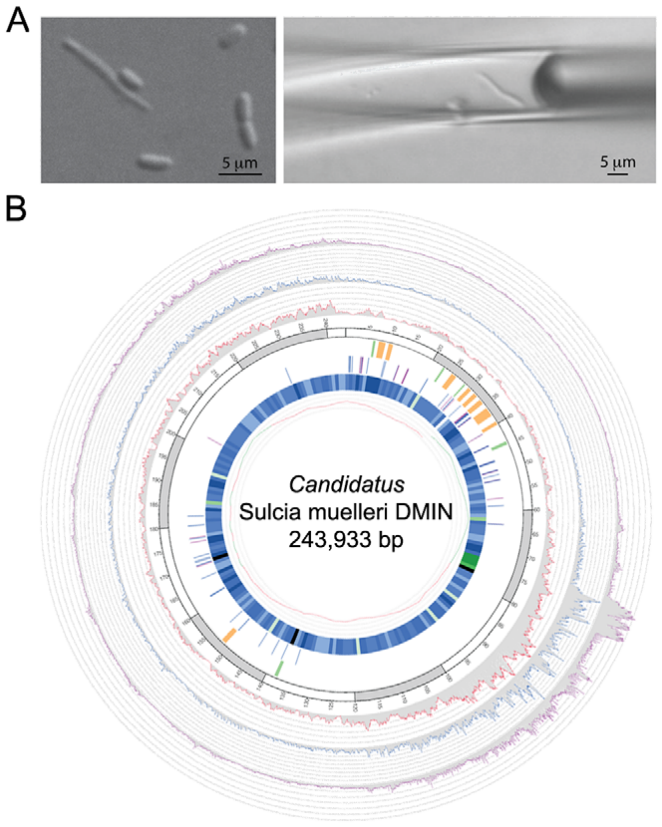
*Sulcia* cell isolation and sequence coverage, closure and polishing locations along the *Sulcia* DMIN single cell genome. (A) Micromanipulation of the single *Sulcia* cell from the sharpshooter bacteriome metasample. (B) Sequence coverage including closure and polishing locations along the finished, circular *Sulcia* DMIN genome with circles corresponding to following features, starting with outermost circle: (1) Illumina sequence coverage ranging from 0–3276 (mean 303+−386), (2) pyrosequence sequence coverage ranging from 0–231 (mean 42+−39), (3) Sanger sequence coverage ranging from 0–30 (mean 10+−7), (4) locations of captured (green) and uncaptured gaps (orange), (5) polishing locations corrected using Illumina (blue) and Sanger (purple) seqeunce, (6) GC content heat map (dark blue to light green = low to high values) and (7) GC skew.

*Sulcia* cells have shown to be of distinct strap-like shape with a length of up to 100µm [22]. Using this morphology as a guideline, a single *Sulcia* cell was isolated from the host bacteriome using micromanipulation (Figure 1A), its genome amplified via multiple displacement amplification and sequenced using a combination of Sanger sequence and pyrosequencing, generating a total of 57 Mbp (megabasepairs) of sequence (see Material and Methods for more details). Approximately 90% of the reads were identified as likely contaminants as based on phylogenetic assignments using blastx and MEGAN [29] (Figure S2), as well as by mapping the reads to the previously sequenced genome of *Sulcia* derived from GWSS [23], [24]. Contaminating reads were found to originate from *Delftia acidovorans* as well as likely host. We were unable to detect these non-target DNA molecules prior to shotgun sequencing using rDNA PCR, as no 16S or 18S rRNA genes were found in the contaminating DNA fragments. 16S rDNA PCR libraries created from the SAG MDA (multiple displacement amplification) DNA were thus solely composed of *Sulcia* clones, while our universal 18S rRNA primers did not yield amplicons. Mapping all reads against the previously sequenced GWSS *Sulcia* genome [23], [24] (87.4% sequence identity on the nucleotide level) allowed us to generate a contamination-free *Sulcia* read set, which was assembled resulting in a draft genome of 31 contigs totaling 244,954 bp (see Methods for more details). Mis-assemblies in the draft genome caused by chimeric clones were corrected manually and Sanger sequencing of PCR products, as well as Sanger primer walks off the 3Kbp clones joined the remaining 15 gaps. Polishing was accomplished using a combination of Sanger sequencing of PCR products and Illumina sequencing. We corrected 100 polishing targets using Illumina sequence, while 37 had to be resolved via directed Sanger sequencing within the closed single cell genome. The genome size of the resulting finished *Sulcia* genome is 243,933 bp (Figure 1B, Table 1, Figure S3). The sequence coverage along the genome shows slight variations between the different sequencing platforms, likely arising from platform-specific biases (for an example, [30]). The overall uneven representation is attributed to MDA bias as has been described for other single cell genomes [17], [18]. Interestingly, the genomic region that is most readily covered by 2^nd^ generation sequence data is the region of highest percent GC (Figure 1B), suggesting a possible GC-dependent amplification bias.

**Table 1:** General features of the *Candidatus* Sulcia muelleri DMIN genome.

**Genome statistics**	
Genome size [bp]	243,933
DNA copding bases [bp]	237,536
GC content [%]	22.5
**Gene predictions**	
Total genes	261
Protein coding genes	226
with function prediction	210
w/o function prediction	16
Number of rRNA operons	1
Number of tRNA genes	31

### Genome features

The GSS *Sulcia* genome is 243,933 bp with 261 total predicted genes, 226 of which are predicted coding genes (Table 1). It encodes one ribosomal operon (23S, 16S, and 5S), and 31 tRNAs. The coding density (97%) is among the highest in the Bacteroidetes and among the highest in Bacteria. *Sulcia* has a minimal set of proteins for transcription and translation and the same metabolic capacity as *Sulcia* from GWSS and cicada. The principal role of *Sulcia* appears to be the production of essential amino acids as evidenced by the presence of nearly complete pathways for biosynthesis of lysine (one gene, *dapE*, is missing), leucine, valine, threonine, isoleucine, phenylalanine and tryptophan [23], [24].

Differences in gene content between the DMIN and GWSS *Sulcia* genomes are minimal. A comparison of the two sharpshooter *Sulcia* genomes revealed that SMGWSS_009, a predicted N6-adenine-specific methylase in DMIN *Sulcia*, has been deleted from the GWSS *Sulcia* genome. In addition, two fusion events are present in DMIN *Sulcia* but not in GWSS *Sulcia*. The first event corresponds to the fusion of two ribosomal proteins L10 and L12 (DMIN_00550). The second fusion event is observed between ribosomal protein S21 and the leader peptidase *lepB* (DMIN_01740). The sequence of these regions has been verified with Sanger, 454 and Illumina sequencing and in either case the observed fusion is supported. Neither fusion has been reported from other organisms thus far. High numbers of fusion events have been associated with small genome size [31] and efficiency in transcription or translation [32].

**Figure 2:**
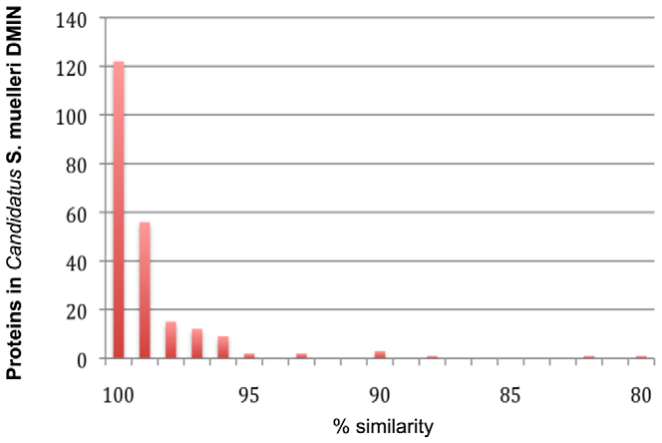
Distribution of protein similarities between the two available genomes of *Candidatus* Sulcia muelleri from sharpshooters.

One interesting question arising from the study of these organisms is the mechanism they use to recognize and interact with the host. We looked for proteins whose similarity between the two genomes is low (Figure 2, Table 2). Among these proteins DMIN_01600, homolog of SMGWSS_162 exhibits similarity to bacterial surface antigens. Homologs of these proteins participate in outer membrane protein complexes of gram-negative bacteria. The location of this protein and its function suggest a role in the bacterial cell – host interaction.

**Table 2:** Pairs of proteins that have low sequence similarity between *Candidatus* S. muelleri DMIN and *Candidatus* S. muelleri GWSS.

*S. muelleri* DMIN	*S. muelleri* GWSS	Protein	Sequence similarity [%]
DMIN_01680	SMGWSS_171	30S ribosomal subunit protein S18	75.34
DMIN_01600	SMGWSS_162	putative outer membrane protein	81.23
DMIN_01330	SMGWSS_137	chaperone protein DnaJ	87.14
DMIN_02500	SMGWSS_255	dihydrolipoamide acyltransferase E2 component	89.05
DMIN_00160	SMGWSS_017	putative ATP synthase F1, epsilon subunit	89.11
DMIN_01860	SMGWSS_191	hypothetical protein	89.71
DMIN_00320	SMGWSS_033	highly suspect ATP synthase F1, delta subunit	92.73
DMIN_02450	SMGWSS_249	putative delta-1-pyrroline-5-carboxylate dehydrogenase	92.74
DMIN_00970	SMGWSS_101	chaperone protein DnaJ	94.13
DMIN_01120	SMGWSS_116	putative acetylornithine deacetylase (ArgE)/succinyl-diaminopimelate desuccinylase (DapE)	94.52
DMIN_00050	SMGWSS_006	phenylalanyl-tRNA synthetase beta subunit	95.04
DMIN_01320	SMGWSS_136	50S ribosomal subunit protein L19	95.29
DMIN_01570	SMGWSS_160	tryptophanyl-tRNA synthetase	95.3
DMIN_01250	SMGWSS_129	ATP-dependent protease	95.32
DMIN_01340	SMGWSS_138	1,4-dihydroxy-2-naphthoate octaprenyltransferase	95.35
DMIN_00300	SMGWSS_031	ATP synthase F1, gamma subunit	95.44
DMIN_02510	SMGWSS_256	integral membrane protein TerC family	95.74
DMIN_01940	SMGWSS_199	translation initiation factor IF-2	95.79

### Sequence variation within *Sulcia* populations

We mapped all single cell Illumina and 454 sequence reads against the completed *Sulcia* genome and found no sequence polymorphisms.

To verify the accuracy of the single cell genome and exclude MDA artifacts such as chimeric rearrangements, we independently sequenced the *Sulcia* genome from a pool of 25 adult hosts using the metagenomic approach. The two sharpshooter samples were collected from field populations in California, maintained in the laboratory, and sampled at different time points. Thus, the nucleotide polymorphisms represent population variation. We were able to identify four regions with such polymorphisms. Beyond these four variations, the two genomes were identical. In two cases the polymorphic sites are located inside CDS regions (DMIN_01600 and DMIN_02500) and consist of differences in numbers of short repeats. In the case of DMIN_01600, we observe either 8 or 9 repeats of a 6 nucleotide sequence. In DMIN_02500, the number of repeats varies between 12 and 17 for a 9 nucleotide sequence; in some cases, this is interrupted by a different 9 nucleotide region (Table 3). For DMIN_02500, the number of repeats also varies between the single cell genome and the single host metagenome, for which we sequenced these four sites (see Material and Methods for more information). Interestingly, DMIN_1600 is predicted to be an outer membrane protein, and this polymorphism could involve interactions with the host cell.

**Table 3:** Sequence variation detected between the single cell genome, the single bacteriome derived metagenome (SH, single host) and the pooled bacteriome derived metagenome (MH, multiple hosts).

Genomic locus		Sequence variation
**DMIN_01600 (outer membrane protein)**	Single cell genome/Metagenome (SH)	TGAA[ttatca]_8_TTAA
	Metagenome (MH)	TGAA[ttatca]_9_TTAA
**DMIN _02500 (dihydrolipoamide acyltransferase E2 component)**	Single cell genome	CTAA[tgaagttaa]_12_ccattctaa[tgaagttaa]_2_ccattctaataTTA
	Metagenome (SH)	CTAA[tgaagttaa]_14_ccattctaa[tgaagttaa]_2_ccattctaataTTA CTAA[tgaagttaa]_17_ccattctaataTTA
	Metagenome (MH)	CTAA[tgaagttaa]_14_ccattctaa[tgaagttaa]_2_ccattctaataTA
**DMIN _02080–DMIN _02090**	Single cell genome/Metagenome (SH)	CTTG[ctactta]_16_ATAA
	Metagenome (MH)	CTTG[ctactta]_18_ATAA
**23S rRNA gene**	Single cell genome/Metagenome (SH)	TAAA[agaaattgttgcgaataataaa]_2_[agaaattgagaagttgcgaataataaa]_4_TTTA
	Metagenome (MH)	TAAA[agaaattgttgcgaataataaa]_2_agaaattgagaagttgcgaataataaa[agaaattgttgcgaataataaa]_2_agaaattgagaagttgcgaataataaaTTTA

Lastly, we aimed to evaluate the degree of heterogeneity within the *Sulcia* population by sequence analysis of *Sulcia* metagenomic reads derived from (i) the single sharpshooter bacteriome of which the single cell genome originated and for which we generated 454 and Illumina data, and (ii) the pool of 25 sharpshooter bacteriomes, which lead to the metagenome-derived *Sulcia* genome, for which we had generated 454 data (see Material and Methods for more detail). Based on simulated datasets (Figure S4), we estimate that 20× depth is sufficient to identify ∼90% of all SNPs at allele frequency 0.5 and ∼60% of all SNPs at allele frequency 0.25. Given that both metagenomic datasets covered >67% of the *Sulcia* genome at a minimum depth of 20× (Figure S4, see Material and Methods for more details), we estimate that we would have found ∼60% of all SNPs at allele frequency 0.5 and 40% of all SNPs at allele frequency 0.25. No potential SNPs were identified in the single host metagenome, while we identified two SNPs in the host pool metagenome, in genes DMIN_00390 and DMIN_01310 (Table 4), indicating that the genetic diversity of this spatially defined *Sulcia* population within its culture-maintained host is very low.

**Table 4:** Identified SNPs within the host pool metagenomic sequence reads.

Genomic locus		Sequence variation
**DMIN_00390 (3-isopropylmalate dehydratase, small subunit)**	Single cell genome	TTTTTTCAAAAATTTC[ta]TTTTTTTTTT
	Metagenome (MH)	TTTTTTCAAAAATTTC[ag]TTTTTTTTTT
**DMIN_01310 (protein translocase subunit secA)**	Single cell genome	ATCAACTCTTT[a]CCCATATAA
	Metagenome (MH)	ATCAACTCTTT[c]CCCATATAA

Genomic variation is a function of the product of the mutation rate and the coalescence time of the sequenced genomes. Coalescence time is likely very short for genome copies within a cell, due to bottlenecks at cell division and during transmission between host generations. For example, studies on genome variation in *Buchnera* of pea aphids found complete lack of variation among genome copies within a single lab colony of aphids, and about 0.3% sequence divergence over an estimated period of about 20,000 years [33]. Indeed *Sulcia* in sharpshooters has been found to have a relatively slow rate of sequence evolution based on comparisons with *Baumannia cicadellinicola*, a co-diverging symbiont in the same hosts [34]. Our results show that our methods do detect actual polymorphism, but that the level of polymorphism was low, at least for our samples.

### Summary

While the current single cell approach leaves room for improvement with respect to the elimination of exogenous DNA contamination and reduction of the amplification bias, this study represents a proof-of-principle for the reconstruction of high quality, finished single cell genomes from uncultured, environmental species. When accessing the DMIN *Sulcia* genome using metagenomics, pooling of the bacteriome of approximately 25 adult hosts is necessary to obtain sufficient DNA for metagenome shotgun sequencing, while only a single cell from a single host bacteriome is required using the single cell approach. Heterogeneity in metagenomic samples can lead to the assembly of composite genomes, while a single cell genome accurately captures each base of a single genome within an individual. It can be used as complete reference genome within a population, to study interspecies and intraspecies population genetic variation when combined with metagenomic sequence data or by comparing several environment-derived single cell genomes of identical 16S rRNA sequence. The power of this approach will not only significantly influence discoveries made in microbial ecology and evolution, but also impact studies deciphering the human-associated microbiota.

## Materials and Methods

### Sample origin

Adult green sharpshooter (GSS) *Draeculacephala minerva* were obtained from laboratory cultures (Rodrigo Almeida, UC Berkeley) initially established from insects that were wild-caught in and around Berkeley, CA.

### Single cell digital PCR

Digital PCR was used to determine the genome copy numbers of individual *Sulcia* cells. The yellow portion of the bacteriome was dissected and resuspended in PBS and symbiont cells freed using tituration. Single cells were then isolated using an Axio Observer D1 inverted microscope (Zeiss) and TransferMan NK2 micromanipulator with a cell tram vario (Eppendorf). Four single *Sulcia* cells captured in PBS were subjected to shearing using a Corvaris S2 (Corvaris) to approximately 3Kb fragments (Duty Cycle = 20%, Intensity = 0.1, 1,000 cycles per burst, 300 seconds). Two *Sulcia*-specific primer sets were designed (see Table S1). The primers were tested using 1ul of the sheared single cell lysates that was MDA-amplified to verify the correct product length and product specificity. 1ul of sheared single cell lysates was then subjected to digital PCR using the *Sulcia*-specific primer sets and according to the manufacture protocol (Fluidigm).

### Single cell MDA and genome sequencing

The yellow portion of the bacteriome of a single GSS collected in January 2008 was dissected, resuspended in PBS and symbiont cells freed using tituration. Single cells were then isolated as described above. An individual *Sulcia* cell was lysed and amplified using the Repli-g UltraFast Mini Kit (Qiagen) according to the manufacturer's instructions, but increasing amplification duration to 16 hours. Shotgun sequencing using Sanger and pyrosequencing was performed for the SAG as follows. MDA products were debranched using S1 nuclease (Fermentas) digestion and 3 Kbp Sanger libraries, as well as 454 libraries, constructed as described previously [17]. Sanger clones were sequenced on an ABI PRISM 3730 capillary DNA sequencer (Applied Biosystems) according to the JGI standard protocols (www.jgi.doe.gov) yielding 6,144 raw reads. Quality trimming resulted in 5,546 reads (>/ = Q20) totaling 3.43 Mbp of sequence. Pyrosequencing was performed using the Genome Sequencer FLX System (Roche/454) [35] according to the manufacturer protocol generating 231,073 reads totaling 47 Mbp of sequence.

### Sequence read analysis, assembly and finishing of the *Sulcia* SAG

The single cell genome sequence reads were analyzed using BLASTX (e-10, 10 best hits) and lowest common ancestor algorithm (LCA) assignments using MEGAN [29], as well as GC content analysis, detecting high levels of contaminating reads (Figure S2). We identified these non-target reads to largely originate from *Delftia acidovorans*, as well as likely from the host. The contamination had not been detectable by 16S rDNA or 18S rDNA PCR libraries, as these genes were not encoded in any of the contaminating DNA fragments. *D. acidovorans* DNA was found to be reagent-derived (unpublished data), while the free host DNA was likely introduced into the sample during micromanipulation. Due to MDA bias causing uneven amplification of genomic regions, we were unable to bin the target genome reads as based on GC contents and sequence depth. Contaminating reads were identified and removed by aligning all of the reads to the reference genome (NC_010118.1; *Candidatus* Sulcia muelleri GWSS, complete genome) using Newbler mapper (Newbler version 2.0.0-PostRelease-09/05/2008, Roche/454), which left us with 1,248 Sanger reads and 38,532 454 reads that were target genome specific. The *Sulcia* pyrosequence was assembled using the 454 Newbler assembler version 2.0.0-PostRelease-09/05/2008 (Roche/454) and the consensus sequence shredded into 1 Kbp pieces with 100 bp overlaps. The 454 shred data was then assembled with the contamination screened Sanger sequences using lucyPGA (lucy version 1.19p [36], Paracel Genome Assembler 2.6.2, Paracel, Pasadena, CA) resulting in a draft assembly of 31 contigs totaling 244,954 bp. Where possible, Newbler assembly sequence was added manually and gaps in the draft were joined manually. This was largely in regions where the PGA assembler had broken the assembly due to chimeric clones. Such misassemblies explain the increased draft genome size as compared to the final finished product. The remaining 15 gaps were then closed by Sanger sequencing of PCR products as well as Sanger primer walking using the 3Kbp clones. After genome closure, unpolished genomic regions were identified using polisher/acePolisher programs [37]. Illumina libraries were constructed from the *Sulcia* SAG DNA according to the manufacturer's instructions and 16,905,451 reads totaling 705,281,500 bp were generated on the Genome Analyzer II. One hundred polishing targets were successfully resolved by alignment of the Illumina sequence data. An additional 37 genomic regions had to be polished by PCR and Sanger sequencing. The remaining identified polishing targets were unsupported. For all PCRs, the failsafe PCR kit (Epicentre) was used. The final assembly yielded the finished *Sulcia* genome of 243,929 bp.

### 16S rRNA clone libraries

Bacterial 16S rDNA PCR libraries were created for the single cell MDA product using primers 27f and 1391r, as described previously [17]. 96 clones were sequenced per library using an ABI PRISM 3730 capillary DNA sequencer (Applied Biosystems). The bi-directional 16S rDNA sequence reads were end-paired, trimmed for PCR primer sequence and quality, and analyzed using BLASTN (Altschul, 2001). For the SAG, ribosomal RNA gene PCR amplification using universal archaeal 16S primers as well as eukaryotic 18S primers was attempted but did not yield any PCR products.

### Single host bacteriome metagenome sequencing

The cell material remaining from the bacteriome the single cell genome was isolated from, was lysed and amplified as described for the single cells. To minimize the amplification bias, we amplified 20 aliquots of cell material. PCR products were pooled for Illumina library construction according to the JGI standard protocols (www.jgi.doe.gov). Illumina sequencing [38] was performed on the metagenome using the Genome Analyzer II System according to the manufacturer's specifications generating 24,446,063 reads totaling ∼1,8 Gbp of sequence. We moreover generated pyrosequence using the Genome Sequencer FLX System (454 Life Sciences, http://www.454.com/) [35] with long-read GS FLX Titanium chemistry according to the manufacturer protocol generating 504,581 reads totaling 195 Mbp of sequence.

### Pooled host bacteriome metagenome sequencing

Bacteriomes of approximately 25 female GSS were collected in August 2007 in Berkeley, CA on Bermuda grass, dissected in 1× PBS, and transferred to 95% ethanol for storage. DNA was purified from these dissected bacteriomes using the Qiagen DNeasy Blood & Tissue kit. The purified DNA was prepared and sequenced on a Roche 454 FLX DNA sequencer, as directed by the manufacturer, at the University of Arizona Genetics Core facility. The 454 run generated 230,307 reads totaling 57,411,340 nts, and these reads were assembled into 407 contigs greater than 500 nts using version 1.1.02.15 of the Newbler assembler. The previously published *Sulcia* genome from GWSS [24] was used in BLASTN searches to identify contigs comprising the GSS *Sulcia* genome. Nine contigs totaling 242,254 nts were identified as putatively belonging to the *Sulcia* genome, and this was confirmed by joining the contigs into a circular genome by PCR and Sanger sequencing. The GWSS *Sulcia* genome was used to correct a large number of homopolymer errors introduced by 454 sequencing. Alignments were made between the GWSS and GSS genomes using BLASTN (with filtering turned off, −F F), and the GSS sequence was adjusted if the region of alignment was high quality, the homopolymer length was greater than 4 nts and differed by only one nt. Any remaining errors in coding regions were verified or corrected using PCR and Sanger sequencing. This procedure is therefore expected to leave uncorrected homopolymer errors in intergenic regions.

### Sequence analysis and annotation

The gene modeling program Prodigal (http://prodigal.ornl.gov/) was run on the finished *Sulcia* single cell genome, using default settings that permit overlapping genes and using ATG, GTG, and TTG as potential starts. The resulting protein translations were compared to Genbank's non-redundant database (NR) the Swiss-Prot/TrEMBL, PRIAM, Pfam, TIGRFam, Interpro, KEGG, and COGs databases using BLASTP or HMMER. From these results, product assignments were made. Initial criteria for automated functional assignment set priority based on PRIAM, TIGRFam, Pfam, Intepro profiles, pairwise BLAST vs Swiss-Prot/TrEMBL, KEGG, and COG groups. Manual corrections to automated functional assignments were completed on an individual gene-by-gene basis as needed. The annotation was imported into The Joint Genome Institute Integrated Microbial Genomes (IMG; http://img.jgi.doe.gov/cgi-bin/pub/main.cgi) [39].

### Variation detection between the single cell and metagenome derived *Sulcia*

We aligned the metagenome derived *Sulcia* genome against our SAG using QTL aligner. We detected four polymorphic sites between the two genomes. To evaluate if these polymorphisms also exist within the *Sulcia* population of the single bacteriome of which we derived the single cell genome from, we sequenced the four loci within the metagenomic DNA of the single host bacteriome using Sanger sequencing. While three loci were identical between the single cell genome and the single bacteriome metagenome, one site indicated a variation in the number of repeats (Table 3).

### Single nucleotide polymorphism (SNP) analysis

Polymorphism analysis was performed within the single cell *Sulcia* DMIN genome, as well as between the single cell genome and the two sets of metagenomic *Sulcia* reads.

All Sanger, pyrosequence and Illumina reads generated from the SAG were mapped to the finished single cell genome by vmatch (http://www.vmatch.de/). The vmatch was run for both directions of the query sequences with the following parameter setting “−l 50 −e 3”. Sequence regions containing potential SNPs were extracted and aligned with the corresponding query sequences by MUSCLE for verification. At least 3 occurrences were required for a SNP call in regions of perfect alignments upstream and downstream of the SNP (3 bp/each) and sequence qualities of > = 30. No SNP was identified.

Reads of both metagenomic data sets (single bacteriome and bacteriomes of 25 pooled sharpshooter hosts) were aligned to the finished single cell *Sulcia* genome using Cross_match to identify potential SNPs using Consed (www.phrap.org). For the single bacteriome metagenome, 544,859 reads totaling 47 Mbp sequence mapped to the *Sulcia* DMIN genome. For the bacteriome pool metagenome, 64,206 reads totaling 17 Mbp of sequence mapped to the *Sulcia* DMIN genome. Since the single bacteriome metagenomic DNA was MDA-derived, there was a high variability of metagenomic read depth along the *Sulcia* genome with 99.8% and 93% of the genome covered at ≥3×, 87% and 89% of the genome covered at ≥10×, 67% and 68% of the genome covered at ≥20×, and 34% and 10% of the genome covered at ≥40×. Given this level of coverage and based on simulations of pooled data (Figure S4), we conclude that we would have found ∼60% of all SNPs in the *Sulcia* population at allele frequency 0.5 and ∼40% at allele frequency 0.25. Potential SNPs were located by identifying positions that had at least 3 reads of q25 or greater that did not match the reference. Each potential SNP was then manually verified.

### Accession Numbers

The sequence data has been deposited in GenBank (http://www.ncbi.nlm.nih.gov/Genbank) under project accession CP001981 (*Candidatus* Sulcia muelleri DMIN).

## Supporting Information

Figure S1 Digital PCR on *Sulcia* single cell genomes. (A) Single *Sulcia* DMIN cells 1–4 were isolated using micromanipulation to determine the genome copy numbers using digital PCR. Cells were viewed with a LD A-Plan 20× objective, Optovar 2.5×, DICT TL Phase 3. (B) Images of microfluidic digital PCR chips results. Approximately 5% of original sheared single cell material was loaded into the microfluidic chip for amplification. This led us to count 180 genome copies for cell 1 (both loci), 140 (locus A) respectively 240 (locus B) genome copies for cell 2, 740 (locus A) respectively 880 (locus B) genome copies for cell 3, and 480 genome copies for cell 4 (both loci).
(2.84 MB TIF)

Figure S2 GC content of sequence reads. Reads were binned as based on blastx and phylogenetic assignments using lowest common ancestor algorithms in MEGAN. Approximately 35% of the reads were assigned to Proteobacteria while ∼43% could not be assigned due to the lack of BLASTX ‘hits’ in NCBI. Many of the proteobacteria-related reads could be identified as *Delftia acidovorans* with 97–100% nucleotide-level identity to the published *D. acidovorans* SPH-1 genome. The read bin without assignment may largely contain reads from the host insect, which has not been sequenced. Approximately 11% of the reads were assigned to the phylum of Bacteroidetes, representative of the *Sulcia* genome reads. The remaining 11% of the reads were either assigned to Eukarya (∼3%), other bacterial phyla (∼1%) or to other tree nodes higher than phylum level (∼7%).
(8.82 MB TIF)

Figure S3 Circular view of the *Candidatus* Sulcia muellerii DMIN genome. Circles correspond to following features, starting with outermost circle: (1) genes on forward strand (color by COG categories), (2) genes on reverse strand (color by COG categories), (3) RNA genes (tRNAs green, sRNAs red, other RNAs black), (4) GC content and (5) GC skew.
(0.65 MB TIF)

Figure S4 Estimated SNP recovery rates at given sequence depths, based on simulated *Escherichia coli* datasets. Reads from two strains of *E. coli* were combined, generating a series of data sets that varied in both depth and ratio of contribution from each strain. To simulate allele frequency of .25 and read depth 40×, reads totaling 30× of average read depth for strain A and 10× for strain B were randomly selected and aligned to strain A's reference. The percentage of the known 170 variants between the two strains that were correctly identified using consed are reported. Using the above simulations and the metagenome coverage (>67% of their genomes covered at a minimum depth of 20×), we estimate that we have found ∼60% (67% coverage×90% SNP discovery rate) of all SNPs at allele frequency 0.5 and 40% (67% coverage×60% SNP discovery rate) of all SNPs at allele frequency of .25.
(8.05 MB TIF)

Table S1 Primers and probes used for DMIN *Sulcia* single cell dPCR.
(0.04 MB DOC)
