# Supplementary figures and images for: One Bacterial Cell, One Complete Genome
Source: PLoS One. 2010 Apr 23;5(4):e10314. doi: 10.1371/journal.pone.0010314 (PMC2859065; doi:10.1371/journal.pone.0010314)

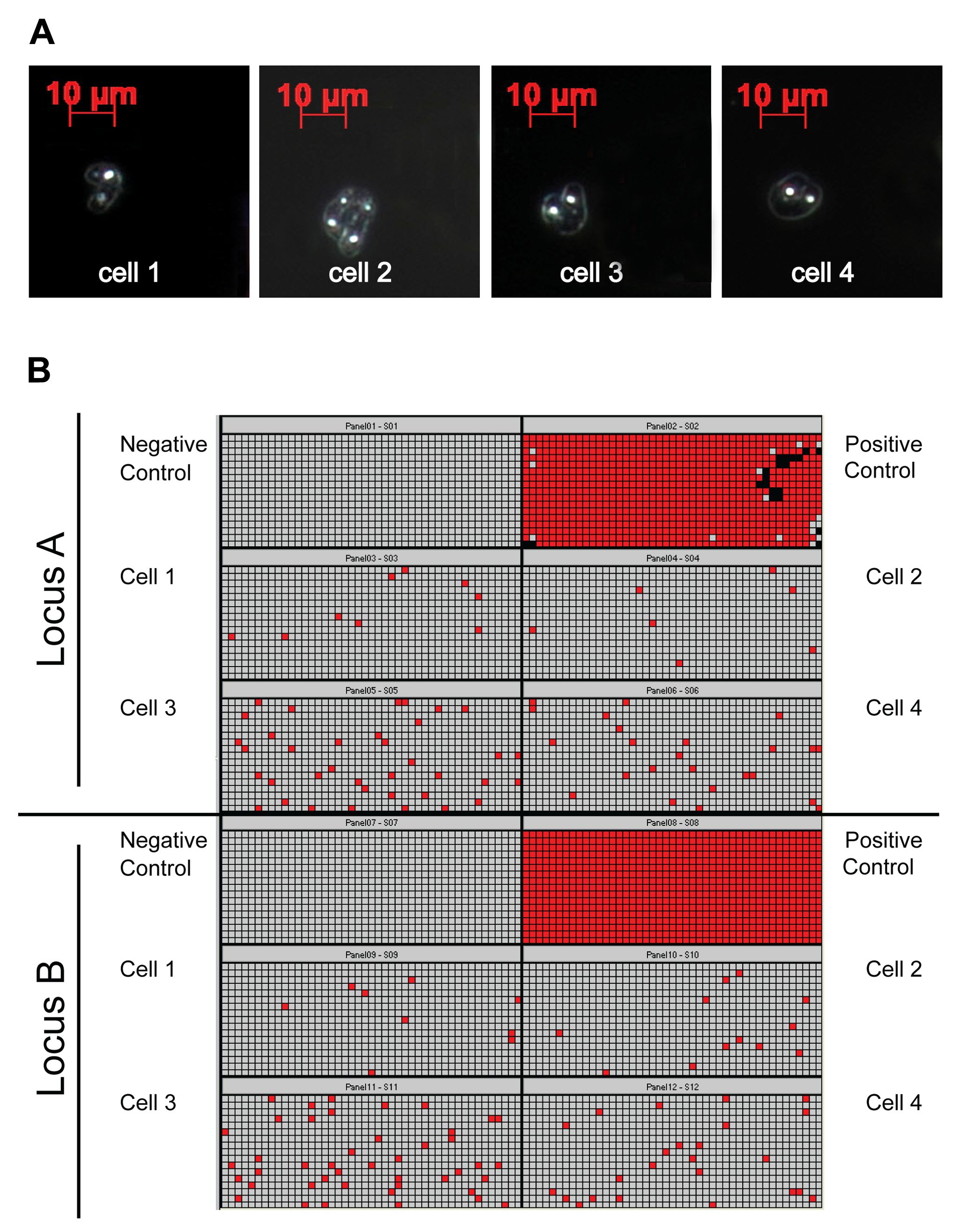

Supplement: Figure S1 — Digital PCR on Sulcia single cell genomes. (A) Single Sulcia DMIN cells 1–4 were isolated using micromanipulation to determine the genome copy numbers using digital PCR. Cells were viewed with a LD A-Plan 20× objective, Optovar 2.5×, DICT TL Phase 3. (B) Images of microfluidic digital PCR chips results. Approximately 5% of original sheared single cell material was loaded into the microfluidic chip for amplification. This led us to count 180 genome copies for cell 1 (both loci), 140 (locus A) respectively 240 (locus B) genome copies for cell 2, 740 (locus A) respectively 880 (locus B) genome copies for cell 3, and 480 genome copies for cell 4 (both loci). (2.84 MB TIF) [file pone.0010314.s001.tif]

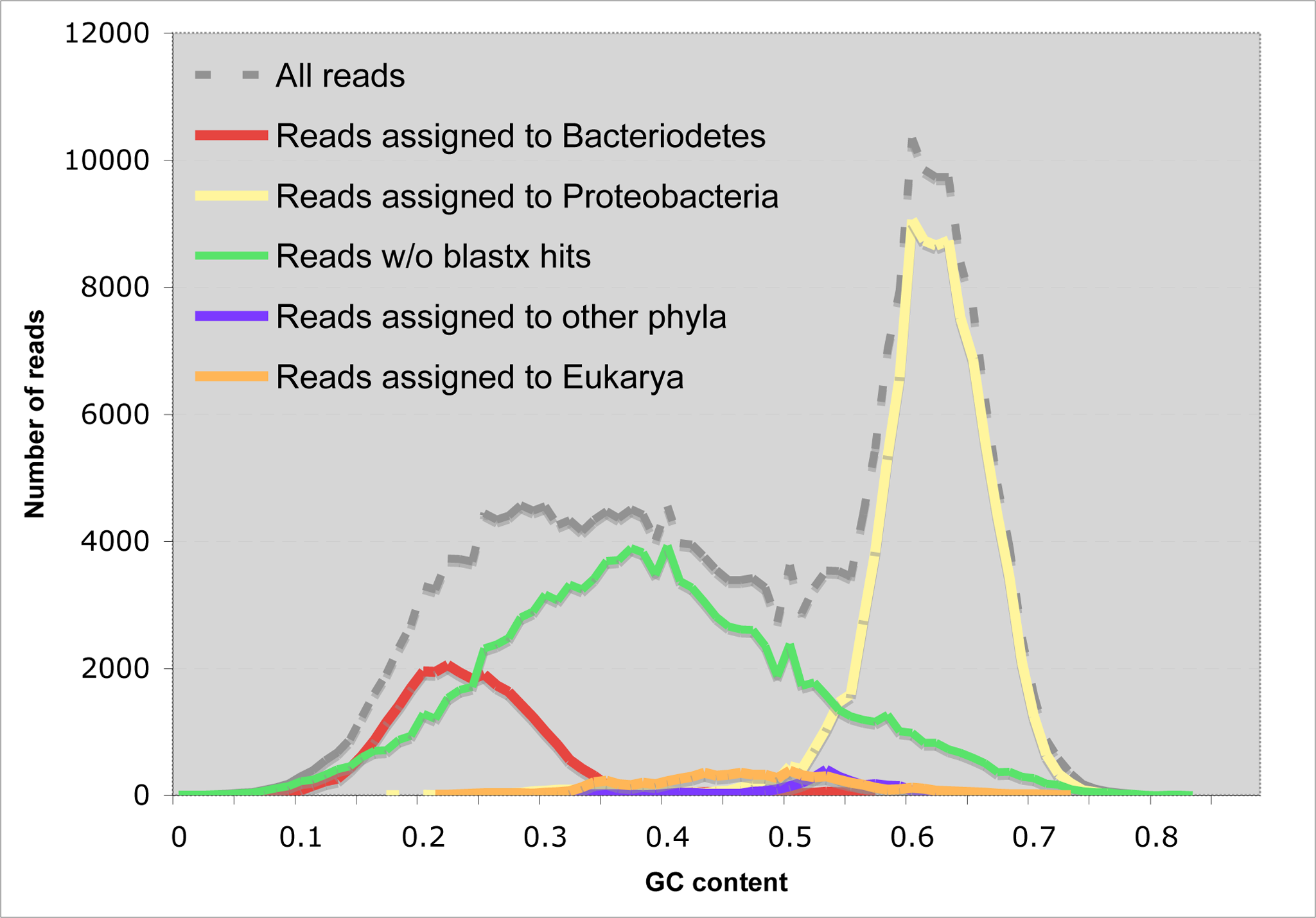

Supplement: Figure S2 — GC content of sequence reads. Reads were binned as based on blastx and phylogenetic assignments using lowest common ancestor algorithms in MEGAN. Approximately 35% of the reads were assigned to Proteobacteria while ∼43% could not be assigned due to the lack of BLASTX ‘hits’ in NCBI. Many of the proteobacteria-related reads could be identified as Delftia acidovorans with 97–100% nucleotide-level identity to the published D. acidovorans SPH-1 genome. The read bin without assignment may largely contain reads from the host insect, which has not been sequenced. Approximately 11% of the reads were assigned to the phylum of Bacteroidetes, representative of the Sulcia genome reads. The remaining 11% of the reads were either assigned to Eukarya (∼3%), other bacterial phyla (∼1%) or to other tree nodes higher than phylum level (∼7%). (8.82 MB TIF) [file pone.0010314.s002.tif]

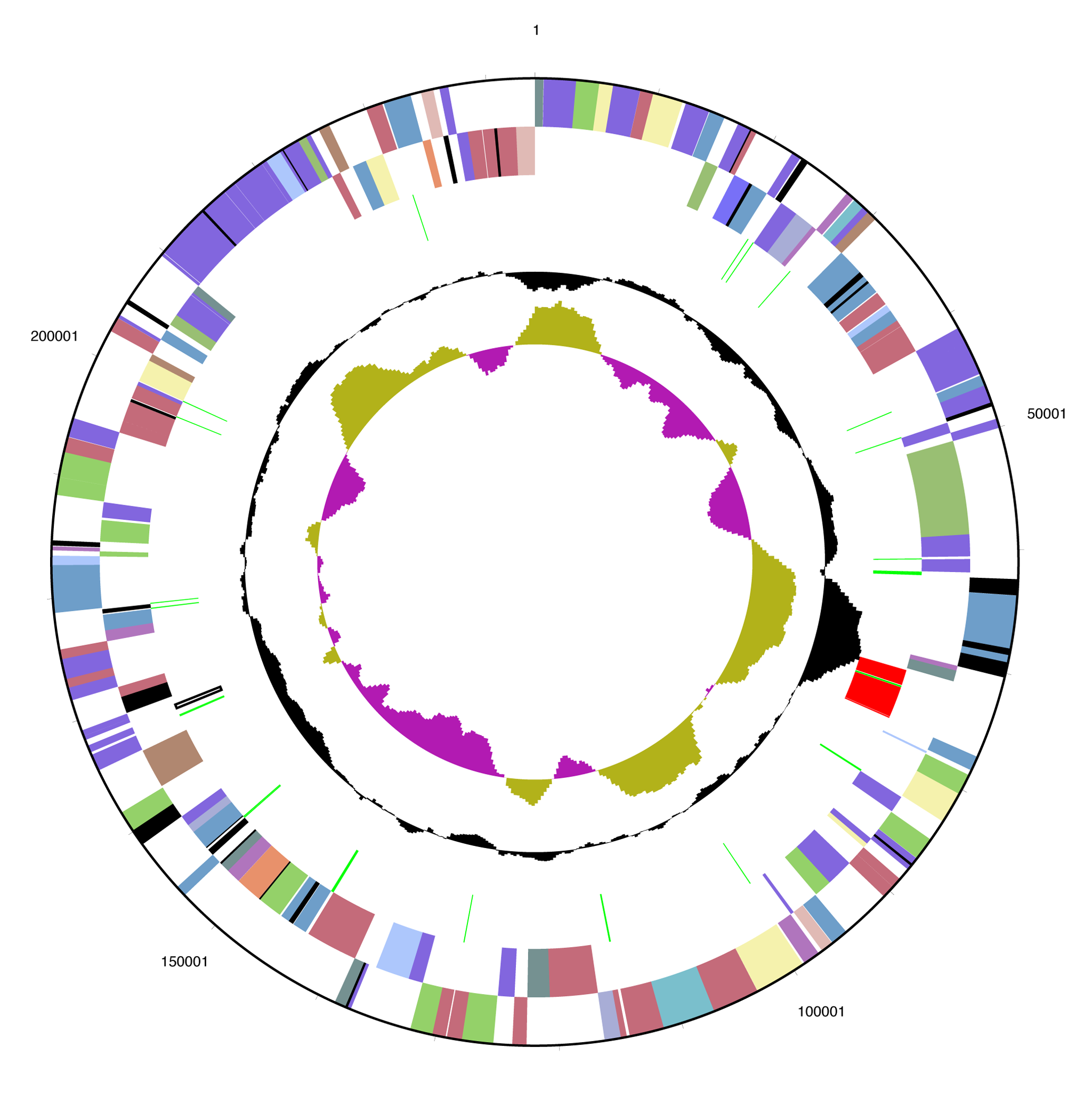

Supplement: Figure S3 — Circular view of the Candidatus Sulcia muellerii DMIN genome. Circles correspond to following features, starting with outermost circle: (1) genes on forward strand (color by COG categories), (2) genes on reverse strand (color by COG categories), (3) RNA genes (tRNAs green, sRNAs red, other RNAs black), (4) GC content and (5) GC skew. (0.65 MB TIF) [file pone.0010314.s003.tif]

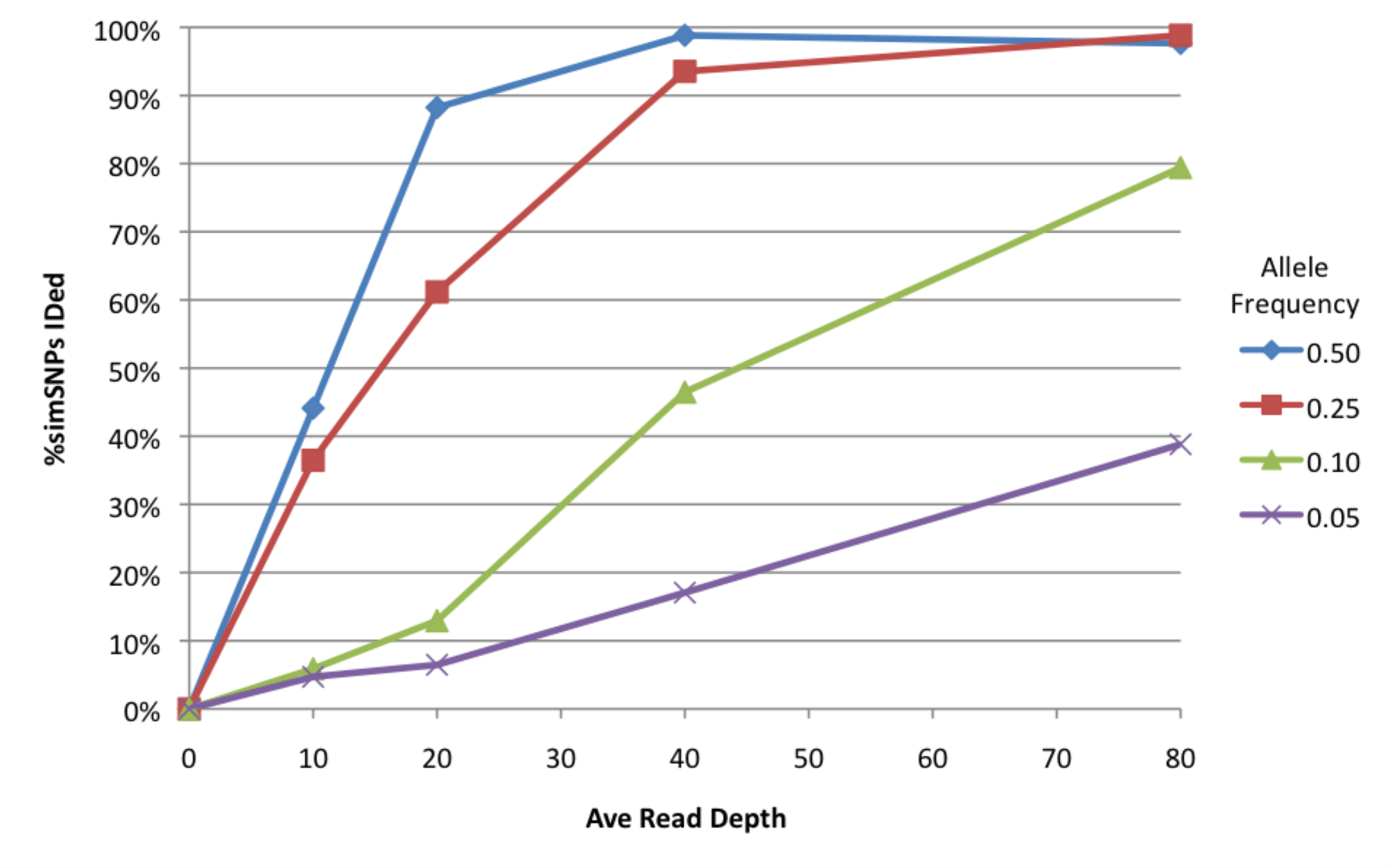

Supplement: Figure S4 — Estimated SNP recovery rates at given sequence depths, based on simulated Escherichia coli datasets. Reads from two strains of E. coli were combined, generating a series of data sets that varied in both depth and ratio of contribution from each strain. To simulate allele frequency of .25 and read depth 40×, reads totaling 30× of average read depth for strain A and 10× for strain B were randomly selected and aligned to strain A's reference. The percentage of the known 170 variants between the two strains that were correctly identified using consed are reported. Using the above simulations and the metagenome coverage (>67% of their genomes covered at a minimum depth of 20×), we estimate that we have found ∼60% (67% coverage×90% SNP discovery rate) of all SNPs at allele frequency 0.5 and 40% (67% coverage×60% SNP discovery rate) of all SNPs at allele frequency of .25. (8.05 MB TIF) [file pone.0010314.s004.tif]
