# Supplementary material for: One Bacterial Cell, One Complete Genome
Source: PLoS One. 2010 Apr 23;5(4):e10314. doi: 10.1371/journal.pone.0010314 (PMC2859065; doi:10.1371/journal.pone.0010314)
Supplement: Table S1 — Primers and probes used for DMIN Sulcia single cell dPCR. (0.04 MB DOC) [file pone.0010314.s005.doc]

**Table S1.** Primers and probes used for DMIN *Sulcia* single cell dPCR.

| **Primer name** | **Primer sequence (5’-3’)** |
| --- | --- |
| Sulcia LocusA_FW**-tag** | **GGC GGC GA**G GGC AGC AGG TGT TAA AGG TGC TAT |
| Sulcia LocusA_FW | GGG CAG CAG GTG TTA AAG GTG CTA T |
| Sulcia LocusA_RV | GCA GGT AGA GCT ATC ATA GGA TGA CCA |
| Sulcia LocusB_FW**-tag** | **GGC GGC GA**A CCG GAC CCT ATA ACA CGT ACC ACA |
| Sulcia LocusB_FW | ACC GGA CCC TAT AAC ACG TAC CAC A |
| Sulcia LocusB_RV | TTC ATT GCT CCA TTA CCT ACA TGC CAA |
